# Supplementary material for: Demography and homing behavior in the poorly-known Philippine flat-headed frog Barbourula busuangensis (Anura: Bombinatoridae)
Source: PeerJ. 2025 Jan 14;13:e18694. doi: 10.7717/peerj.18694 (PMC11740736; doi:10.7717/peerj.18694)
Supplement: Supplemental Information 1 [file peerj-13-18694-s001.docx]

**S1** Output table of the adjusted linear model that explores the relationship between the SVL of individuals at their first capture, and the growth rate calculated upon subsequent recaptures.

| **Predictor** | **Estimate (B)** | **Standard error** | **t-value** | ***p*** |
| --- | --- | --- | --- | --- |
| Intercept | 1.930 | 0.284 | 6.788 | 2.86E-09 |
| SVL | -0.035 | 0.005 | -7.631 | **8.06E-11*** |
| Locality (San Rafael) | -0.138 | 0.080 | -1.706 | 0.0924 |
