# Supplementary material for: Demography and homing behavior in the poorly-known Philippine flat-headed frog Barbourula busuangensis (Anura: Bombinatoridae)
Source: PeerJ. 2025 Jan 14;13:e18694. doi: 10.7717/peerj.18694 (PMC11740736; doi:10.7717/peerj.18694)
Supplement: Supplemental Information 2 — Models are ranked by their AICc value. Apparent survival of individuals is coded as ϕ, p is the probability of capture, and pent is the rate of entrance of new individuals into the study area. A period (.) is used to represent the parameters that are kept constant in the model, and a (t) for parameters that were modelled as time-dependent. [file peerj-13-18694-s002.docx]

**S2** Results of all models generated by the overall POPAN analysis in Malbato and San Rafael across the entire study period. Models are ranked by their AICc value. Apparent survival of individuals is coded as *ϕ*, *p* is the probability of capture, and *pent* is the rate of entrance of new individuals into the study area. A period (.) is used to represent the parameters that are kept constant in the model, and a (t) for parameters that were modelled as time-dependent.

| **Model** | **AICc** | **AICc weight** | **Parameters** | **Deviance** |
| --- | --- | --- | --- | --- |
| **Malbato** |  |  |  |  |
| *Φ(.), p(.), pent(t)* | 2155.49 | 0.999 | 42 | 215 |
| *Φ(.), p(t), pent(t)* | 2169.31 | 0.001 | 80 | 126 |
| **San Rafael** |  |  |  |  |
| *Φ(.), p(t), pent(t)* | 511.79 | 0.754 | 18 | -422.1 |
| *Φ(.), p(t), pent(.)* | 514.12 | 0.235 | 11 | -402.8 |
| *Φ(t), p(t), pent(t)* | 520.44 | 0.01 | 23 | -425.6 |
