# Supplementary material for: Demography and homing behavior in the poorly-known Philippine flat-headed frog Barbourula busuangensis (Anura: Bombinatoridae)
Source: PeerJ. 2025 Jan 14;13:e18694. doi: 10.7717/peerj.18694 (PMC11740736; doi:10.7717/peerj.18694)
Supplement: Supplemental Information 3 — Apparent survival of individuals is given by ϕ, p is the probability of capture, pent the rate of entrance of new individuals in the study area between two sampling occasions and N is the estimated abundance of B. busuangensis. [file peerj-13-18694-s003.docx]

**S3** Estimates for the parameters *ϕ*, *p*, *pent* and *N* for all models with AICc weight > 0.05 from Malbato over our study period. Apparent survival of individuals is given by *ϕ*, *p* is the probability of capture, *pent* the rate of entrance of new individuals in the study area between two sampling occasions and *N* is the estimated abundance of *B. busuangensis*.

| **Model** | **Parameters** | **Estimates (lower–upper 95% CI)** |
| --- | --- | --- |
| *Φ(.), pent(t), p(.)* | *Φ* | 0.996 (0.995–0.997) |
|  | *p* | 0.093 (0.081–0.106) |
|  | *pent* | < 0.001 (0–< 0.001) |
|  | *pent* | < 0.001 (0–< 0.001) |
|  | *pent* | < 0.001 (< 0.001–1) |
|  | *pent* | < 0.001 (0–< 0.001) |
|  | *pent* | < 0.001 (0–< 0.001) |
|  | *pent* | 0.088 (0.023–0.279) |
|  | *pent* | < 0.001 (< 0.001–1) |
|  | *pent* | < 0.001 (< 0.001–< 0.001) |
|  | *pent* | < 0.001 (0–< 0.001) |
|  | *pent* | < 0.001 (0–< 0.001) |
|  | *pent* | 0.127 (0.050–0.286) |
|  | *pent* | < 0.001 (0–< 0.001) |
|  | *pent* | < 0.001 (0–< 0.001) |
|  | *pent* | < 0.001 (< 0.001–< 0.001) |
|  | *pent* | < 0.001 (0–< 0.001) |
|  | *pent* | 0.258 (0.173–0.365) |
|  | *pent* | < 0.001 (0–< 0.001) |
|  | *pent* | < 0.001 (< 0.001–< 0.001) |
|  | *pent* | < 0.001 (< 0.001–1) |
|  | *pent* | < 0.001 (< 0.001–< 0.001) |
|  | *pent* | < 0.001 (0–< 0.001) |
|  | *pent* | < 0.001 (< 0.001–1) |
|  | *pent* | < 0.001 (0–< 0.001) |
|  | *pent* | < 0.001 (0–< 0.001) |
|  | *pent* | < 0.001 (0–< 0.001) |
|  | *pent* | < 0.001 (0–< 0.001) |
|  | *pent* | < 0.001 (0–< 0.001) |
|  | *pent* | 0.181 (0.120–0.263) |
|  | *pent* | < 0.001 (< 0.001–< 0.001) |
|  | *pent* | < 0.001 (0–< 0.001) |
|  | *pent* | < 0.001 (0–< 0.001) |
|  | *pent* | < 0.001 (< 0.001–< 0.001) |
|  | *pent* | < 0.001 (0–< 0.001) |
|  | *pent* | < 0.001 (0–< 0.001) |
|  | *pent* | < 0.001 (< 0.001–1) |
|  | *pent* | < 0.001 (0–< 0.001) |
|  | *pent* | < 0.001 (0–< 0.001) |
|  | *pent* | < 0.001 (0–< 0.001) |
|  | *pent* | < 0.001 (0–< 0.001) |
|  | *N* | 266 (246–296) |
