# Supplementary material for: Demography and homing behavior in the poorly-known Philippine flat-headed frog Barbourula busuangensis (Anura: Bombinatoridae)
Source: PeerJ. 2025 Jan 14;13:e18694. doi: 10.7717/peerj.18694 (PMC11740736; doi:10.7717/peerj.18694)
Supplement: Supplemental Information 4 — Apparent survival of individuals is given by ϕ, p is the probability of capture, pent the rate of entrance of new individuals in the study area between two sampling occasions and N is the estimated abundance of B. busuangensis. [file peerj-13-18694-s004.docx]

**S4** Estimates for the parameters *ϕ*, *p*, *pent* and *N* for all models with AICc weigh > 0.05 from San Rafael over our study period. Apparent survival of individuals is given by *ϕ*, *p* is the probability of capture, *pent* the rate of entrance of new individuals in the study area between two sampling occasions and *N* is the estimated abundance of *B. busuangensis*.

| **Model** | **Parameters** | **Estimates (lower–upper 95% CI)** |
| --- | --- | --- |
| *Φ(.), pent(t), p(t)* | *Φ* | 0.995 (0.992–0.997) |
|  | *p* | 0.999 (< 0.001 –1) |
|  | *p* | 0.152 (0.055–0.357) |
|  | *p* | 0.120 (0.042–0.297) |
|  | *p* | 0.300 (0.206–0.415) |
|  | *p* | 0.484 (0.351–0.619) |
|  | *p* | 0.245 (0.155–0.364) |
|  | *p* | 0.306 (0.183–0.464) |
|  | *p* | 0.153 (0.059–0.342) |
|  | *p* | 0.219 (0.087–0.452) |
|  | *pent* | 0.353 (0.118–0.690) |
|  | *pent* | < 0.001 (< 0.001 –< 0.001) |
|  | *pent* | 0.123 (0.008–0.704) |
|  | *pent* | < 0.001 (< 0.001 –1) |
|  | *pent* | 0.427 (0.206–0.681) |
|  | *pent* | 0.048 (< 0.001 –0.805) |
|  | *pent* | < 0.001 (< 0.001 –1) |
|  | *pent* | < 0.001 (< 0.001 –< 0.001) |
|  | *N* | 233 (200–286) |
| *Φ(.), pent(t), p(.)* | *Φ* | 0.994 (0.992–0.995) |
|  | *p* | 0.307 (0.243–0.380) |
|  | *pent* | 0.084 (0.026–0.236) |
|  | *pent* | < 0.001 (< 0.001 –< 0.001) |
|  | *pent* | 0.298 (0.172–0.463) |
|  | *pent* | 0.129 (0.030–0.409) |
|  | *pent* | 0.214 (0.092–0.423) |
|  | *pent* | 0.114 (0.037–0.301) |
|  | *pent* | < 0.001 (< 0.001 –1) |
|  | *pent* | 0.002 (< 0.001 –1) |
|  | *N* | 228 (199–271) |
