# Supplementary material for: Demography and homing behavior in the poorly-known Philippine flat-headed frog Barbourula busuangensis (Anura: Bombinatoridae)
Source: PeerJ. 2025 Jan 14;13:e18694. doi: 10.7717/peerj.18694 (PMC11740736; doi:10.7717/peerj.18694)
Supplement: Supplemental Information 5 — Apparent survival of individuals is given by ϕ, p is the probability of capture, and pent the rate of entrance of new individuals in the study area between two sampling occasions. A period (.) is used to represent the parameters when kept constant, (t) for parameters that were modelled as time-dependent, (g) for those dependent on age class and (g*t) represents their interaction. [file peerj-13-18694-s005.docx]

**S5** Results of all models generated by the overall POPAN formulation, ranked by the AICc value, of *B. busuangensis* in Malbato during the three sampling seasons (April–June 2022, October–November 2022 and April–June 2023). Apparent survival of individuals is given by *ϕ*, *p* is the probability of capture, and *pent* the rate of entrance of new individuals in the study area between two sampling occasions. A period (.) is used to represent the parameters when kept constant, (t) for parameters that were modelled as time-dependent, (g) for those dependent on age class and (g*t) represents their interaction.

| **Sampling period** | **Model** | **AICc** | **AICc weight** | **Parameters** | **Deviance** |
| --- | --- | --- | --- | --- | --- |
| April–July 2022 | *Φ(.), p(g), pent(g*t)* | 585.38 | 0.955 | 35 | -75.5 |
|  | *Φ(g), p(g), pent (g*t)* | 591.50 | 0.044 | 36 | -72.8 |
|  |  |  |  |  |  |
| October–November 2022 | *Φ(.), p(g), pent(g*t)* | 466.30 | 0.811 | 25 | -120.1 |
|  | *Φ(g), p(g), pent (g*t)* | 469.22 | 0.188 | 26 | -120.1 |
|  | *Φ(t), p(g), pent (g*t)* | 482.81 | 0.00021 | 33 | -128.6 |
|  |  |  |  |  |  |
| April–June 2023 | *Φ(.), p(g), pent(g*t)* | 541.98 | 0.799 | 29 | -56.6 |
|  | *Φ(g), p(g), pent (g*t)* | 544.75 | 0.200 | 30 | -57.0 |
|  | *Φ(t), p(g), pent (g*t)* | 558.41 | 0.00022 | 39 | -73.9 |
|  | *Φ(g), ptg), pent (g*t)* | 564.39 | 0.00001 | 40 | -71.7 |
