# Supplementary material for: Demography and homing behavior in the poorly-known Philippine flat-headed frog Barbourula busuangensis (Anura: Bombinatoridae)
Source: PeerJ. 2025 Jan 14;13:e18694. doi: 10.7717/peerj.18694 (PMC11740736; doi:10.7717/peerj.18694)
Supplement: Supplemental Information 6 — Apparent survival of individuals is given by ϕ, p is the probability of capture, pent the rate of entrance of new individuals in the study area between two sampling occasions and N is the estimated abundance. For all parameters “1” designates subadults and “2” adults. [file peerj-13-18694-s006.docx]

**S6** All estimates for the parameters *ϕ*, *p*, *pent* and *N* for all models with AICc weigh > 0.05 from the site of Malbato during the first sampling season (April–July 2022). Apparent survival of individuals is given by *ϕ*, *p* is the probability of capture, *pent* the rate of entrance of new individuals in the study area between two sampling occasions and *N* is the estimated abundance. For all parameters “1” designates subadults and “2” adults.

| **Model** | **Parameters** | **Estimates (lower–upper 95% CI)** |
| --- | --- | --- |
| *Φ(.), p(g), pent(g*t)* | *Φ* | 0.993 (0.981–0.977) |
|  | *p 1* | 0.228 (0.162–0.310) |
|  | *p 2* | 0.062 (0.038–0.099) |
|  | *pent 1* | < 0.001 (0–< 0.001) |
|  | *pent 1* | 0.078 (< 0.001–0.899) |
|  | *pent 1* | < 0.001 (0–< 0.001) |
|  | *pent 1* | 0.117 (0.005–0.776) |
|  | *pent 1* | < 0.001 (< 0.001–< 0.001) |
|  | *pent 1* | 0.117 (0.005–0.769) |
|  | *pent 1* | < 0.001 (< 0.001–< 0.001) |
|  | *pent 1* | 0.026 (< 0.001–0.999) |
|  | *pent 1* | < 0.001 (< 0.001–< 0.001) |
|  | *pent 1* | 0.200 (0.116–0.322) |
|  | *pent 1* | 0.077 (0.001–0.796) |
|  | *pent 1* | < 0.001 (< 0.001–< 0.001) |
|  | *pent 1* | < 0.001 (0–< 0.001) |
|  | *pent 1* | < 0.001 (0–< 0.001) |
|  | *pent 1* | 0.037 (< 0.001–0.981) |
|  | *pent 2* | 0.209 (0.009–0.879) |
|  | *pent 2* | 0.038 (< 0.001–0.999) |
|  | *pent 2* | < 0.001 (< 0.001–< 0.001) |
|  | *pent 2* | < 0.001 (0–< 0.001) |
|  | *pent 2* | < 0.001 (0–0.004) |
|  | *pent 2* | 0.051 (< 0.001–0.883) |
|  | *pent 2* | < 0.001 (0–< 0.001) |
|  | *pent 2* | < 0.001 (0–< 0.001) |
|  | *pent 2* | < 0.001 (< 0.001–1) |
|  | *pent 2* | < 0.001 (< 0.001–1) |
|  | *pent 2* | < 0.001 (< 0.001–< 0.001) |
|  | *pent 2* | 0.331 (0.169–0.546) |
|  | *pent 2* | < 0.001 (< 0.001–< 0.001) |
|  | *pent 2* | < 0.001 (< 0.001–< 0.001) |
|  | *pent 2* | < 0.001 (< 0.001–< 0.001) |
|  | *N 1* | 28 (24–44) |
|  | *N 2* | 174 (127–256) |
| *Φ(g), p(g), pent(g*t)* | *Φ 1* | 0.996 (0.977–0.999) |
|  | *Φ 2* | 0.988 (0.974–0.994) |
|  | *p 1* | 0.214 (0.148–0.299) |
|  | *p 2* | 0.071 (0.071–0.071) |
|  | *pent 1* | < 0.001 (0–< 0.001) |
|  | *pent 1* | 0.065 (< 0.001–0.978) |
|  | *pent 1* | < 0.001 (0–< 0.001) |
|  | *pent 1* | 0.117 (0.003–0.841) |
|  | *pent 1* | < 0.001 (< 0.001–< 0.001) |
|  | *pent 1* | 0.118 (0.003–0.837) |
|  | *pent 1* | < 0.001 (< 0.001–< 0.001) |
|  | *pent 1* | 0.018 (< 0.001–0.999) |
|  | *pent 1* | < 0.001 (< 0.001–< 0.001) |
|  | *pent 1* | 0.222 (0.016–0.831) |
|  | *pent 1* | 0.053 (< 0.001–0.997) |
|  | *pent 1* | < 0.001 (< 0.001–< 0.001) |
|  | *pent 1* | < 0.001 (< 0.001–< 0.001) |
|  | *pent 1* | < 0.001 (< 0.001–< 0.001) |
|  | *pent 1* | 0.018 (< 0.001–0.999) |
|  | *pent 2* | 0.181 (0.011–0.814) |
|  | *pent 2* | 0.089 (0.001–0.870) |
|  | *pent 2* | < 0.001 (< 0.001–< 0.001) |
|  | *pent 2* | < 0.001 (< 0.001–< 0.001) |
|  | *pent 2* | < 0.001 (< 0.001–< 0.001) |
|  | *pent 2* | 0.094 (0.036–0.224) |
|  | *pent 2* | < 0.001 (< 0.001–< 0.001) |
|  | *pent 2* | < 0.001 (0–0.745) |
|  | *pent 2* | < 0.001 (< 0.001–< 0.001) |
|  | *pent 2* | 0.098 (0.003–0.772) |
|  | *pent 2* | 0.155 (0.017–0.660) |
|  | *pent 2* | < 0.001 (< 0.001–< 0.001) |
|  | *pent 2* | < 0.001 (< 0.001–< 0.001) |
|  | *pent 2* | 0.073 (0.001–0.760) |
|  | *pent 2* | < 0.001 (< 0.001–< 0.001) |
|  | *N 1* | 26 (23–44) |
|  | *N 2* | 180 (132–265) |
