# Supplementary material for: Demography and homing behavior in the poorly-known Philippine flat-headed frog Barbourula busuangensis (Anura: Bombinatoridae)
Source: PeerJ. 2025 Jan 14;13:e18694. doi: 10.7717/peerj.18694 (PMC11740736; doi:10.7717/peerj.18694)
Supplement: Supplemental Information 7 — Apparent survival of individuals is given by ϕ, p is the probability of capture, pent the rate of entrance of new individuals in the study area between two sampling occasions and N is the estimated abundance. For all parameters “1” designates subadults and “2” adults. [file peerj-13-18694-s007.docx]

**S7** All estimates for the parameters *ϕ*, *p*, *pent* and *N* for all models with AICc weigh > 0.05 from the site of Malbato during the second sampling season (October–December 2022). Apparent survival of individuals is given by *ϕ*, *p* is the probability of capture, *pent* the rate of entrance of new individuals in the study area between two sampling occasions and *N* is the estimated abundance. For all parameters “1” designates subadults and “2” adults.

| **Model** | **Parameters** | **Estimates (lower–upper 95% CI)** |
| --- | --- | --- |
| *Φ(.), p(g), pent(g*t)* | *Φ* | 0.969 (0.948–0.982) |
|  | *p 1* | 0.355 (0.256–0.468) |
|  | *p 2* | 0.082 (0.047–0.138) |
|  | *pent 1* | 0.109 (0.002–0.840) |
|  | *pent 1* | 0.166 (0.026–0.591) |
|  | *pent 1* | < 0.001 (0–< 0.001) |
|  | *pent 1* | 0.225 (0.065–0.546) |
|  | *pent 1* | < 0.001 (0–< 0.001) |
|  | *pent 1* | 0.007 (< 0.001–0.999) |
|  | *pent 1* | < 0.001 (0–< 0.001) |
|  | *pent 1* | < 0.001 (0–< 0.001) |
|  | *pent 1* | < 0.001 (0–< 0.001) |
|  | *pent 1* | 0.055 (0.004–0.415) |
|  | *pent 2* | < 0.001 (< 0.001–< 0.001) |
|  | *pent 2* | 0.104 (0.004–0.770) |
|  | *pent 2* | < 0.001 (0–< 0.001) |
|  | *pent 2* | 0.067 (< 0.001–0.954) |
|  | *pent 2* | 0.085 (0.001–0.853) |
|  | *pent 2* | < 0.001 (0–< 0.001) |
|  | *pent 2* | 0.020 (< 0.001–0.998) |
|  | *pent 2* | < 0.001 (0–< 0.001) |
|  | *pent 2* | < 0.001 (0–< 0.001) |
|  | *pent 2* | < 0.001 (0–< 0.001) |
|  | *N 1* | 38 (32–53) |
|  | *N 2* | 184 (128–289) |
| *Φ(g), p(g), pent(g*t)* | *Φ 1* | 0.970 (0.945–0.983) |
|  | *Φ 2* | 0.966 (0.910–0.988) |
|  | *p 1* | 0.354 (0.255–0.467) |
|  | *p 2* | 0.085 (0.042–0.165) |
|  | *pent 1* | 0.108 (0.002–0.848) |
|  | *pent 1* | 0.165 (0.026–0.595) |
|  | *pent 1* | < 0.001 (< 0.001–1) |
|  | *pent 1* | 0.223 (0.066–0.537) |
|  | *pent 1* | < 0.001 (< 0.001–1) |
|  | *pent 1* | 0.008 (< 0.001–0.999) |
|  | *pent 1* | < 0.001 (< 0.001–1) |
|  | *pent 1* | < 0.001 (< 0.001–1) |
|  | *pent 1* | < 0.001 (< 0.001–1) |
|  | *pent 1* | 0.055 (0.004–0.419) |
|  | *pent 2* | 0.009 (0.009–0.009) |
|  | *pent 2* | 0.114 (0.004–0.772) |
|  | *pent 2* | < 0.001 (< 0.001–1) |
|  | *pent 2* | 0.070 (< 0.001–0.947) |
|  | *pent 2* | 0.086 (0.001–0.856) |
|  | *pent 2* | < 0.001 (< 0.001–1) |
|  | *pent 2* | 0.028 (< 0.001–0.983) |
|  | *pent 2* | < 0.001 (< 0.001–1) |
|  | *pent 2* | < 0.001 (< 0.001–1) |
|  | *pent 2* | < 0.001 (< 0.001–1) |
|  | *N 1* | 38 (32–53) |
|  | *N 2* | 186 (128–294) |
