# Supplementary material for: Demography and homing behavior in the poorly-known Philippine flat-headed frog Barbourula busuangensis (Anura: Bombinatoridae)
Source: PeerJ. 2025 Jan 14;13:e18694. doi: 10.7717/peerj.18694 (PMC11740736; doi:10.7717/peerj.18694)
Supplement: Supplemental Information 8 — Apparent survival of individuals is given by ϕ, p is the probability of capture, pent the rate of entrance of new individuals in the study area between two sampling occasions and N is the estimated abundance. For all parameters “1” designates subadults and “2” adults. [file peerj-13-18694-s008.docx]

**S8** All estimates for the parameters *ϕ*, *p*, *pent* and *N* for all models with AICc weigh > 0.05 from the site of Malbato during the third sampling season (April–June 2023). Apparent survival of individuals is given by *ϕ*, *p* is the probability of capture, *pent* the rate of entrance of new individuals in the study area between two sampling occasions and *N* is the estimated abundance. For all parameters “1” designates subadults and “2” adults.

| **Model** | **Parameters** | **Estimates (lower–upper 95% CI)** |
| --- | --- | --- |
| *Φ(.), p(g), pent(g*t)* | *Φ* | 0.976 (0.963–0.985) |
|  | *p 1* | 0.268 (0.182–0.375) |
|  | *p 2* | 0.141 (0.092–0.211) |
|  | *pent 1* | 0.755 (0.364–0.943) |
|  | *pent 1* | < 0.001 (0–< 0.001) |
|  | *pent 1* | 0.021 (< 0.001–0.999) |
|  | *pent 1* | < 0.001 (< 0.001–< 0.001) |
|  | *pent 1* | < 0.001 (0–< 0.001) |
|  | *pent 1* | < 0.001 (0–0.001) |
|  | *pent 1* | 0.136 (0.027–0.464) |
|  | *pent 1* | < 0.001 (< 0.001–< 0.001) |
|  | *pent 1* | 0.086 (0.012–0.423) |
|  | *pent 1* | < 0.001 (< 0.001–< 0.001) |
|  | *pent 1* | < 0.001 (< 0.001–< 0.001) |
|  | *pent 1* | < 0.001 (0–0.028) |
|  | *pent 2* | 0.378 (0.190–0.612) |
|  | *pent 2* | < 0.001 (< 0.001–1) |
|  | *pent 2* | 0.031 (< 0.001–0.923) |
|  | *pent 2* | < 0.001 (< 0.001–1) |
|  | *pent 2* | 0.023 (< 0.001–0.939) |
|  | *pent 2* | 0.023 (0.005–0.093) |
|  | *pent 2* | < 0.001 (< 0.001–1) |
|  | *pent 2* | 0.183 (0.087–0.345) |
|  | *pent 2* | < 0.001 (0–< 0.001) |
|  | *pent 2* | < 0.001 (0–< 0.001) |
|  | *pent 2* | < 0.001 (< 0.001–< 0.001) |
|  | *pent 2* | 0.274 (0.123–0.505) |
|  | *N 1* | 39 (32–57) |
|  | *N 2* | 167 (121–250) |
| *Φ(g), p(g), pent(g*t)* | *Φ 1* | 0.979 (0.962–0.988) |
|  | *Φ 2* | 0.973 (0.951–0.985) |
|  | *p 1* | 0.261 (0.181–0.360) |
|  | *p 2* | 0.149 (0.094–0.228) |
|  | *pent 1* | 0.780 (0.545–0.913) |
|  | *pent 1* | < 0.001 (0–< 0.001) |
|  | *pent 1* | < 0.001 (< 0.001–< 0.001) |
|  | *pent 1* | < 0.001 (< 0.001–< 0.001) |
|  | *pent 1* | < 0.001 (0–< 0.001) |
|  | *pent 1* | < 0.001 (0–< 0.001) |
|  | *pent 1* | 0.135 (0.026–0.478) |
|  | *pent 1* | < 0.001 (0–< 0.001) |
|  | *pent 1* | 0.083 (0.010–0.452) |
|  | *pent 1* | < 0.001 (< 0.001–< 0.001) |
|  | *pent 1* | < 0.001 (< 0.001–< 0.001) |
|  | *pent 1* | < 0.001 (0–< 0.001) |
|  | *pent 2* | 0.367 (0.187–0.593) |
|  | *pent 2* | < 0.001 (< 0.001–1) |
|  | *pent 2* | 0.045 (< 0.001–0.717) |
|  | *pent 2* | < 0.001 (0–< 0.001) |
|  | *pent 2* | 0.027 (< 0.001–0.956) |
|  | *pent 2* | 0.028 (< 0.001–0.913) |
|  | *pent 2* | < 0.001 (< 0.001–1) |
|  | *pent 2* | 0.185 (0.088–0.345) |
|  | *pent 2* | < 0.001 (0–< 0.001) |
|  | *pent 2* | < 0.001 (0–< 0.001) |
|  | *pent 2* | < 0.001 (0–< 0.001) |
|  | *pent 2* | 0.265 (0.119–0.491) |
|  | *N 1* | 38 (31–55) |
|  | *N 2* | 166 (121–247) |
