# Supplementary material for: Demography and homing behavior in the poorly-known Philippine flat-headed frog Barbourula busuangensis (Anura: Bombinatoridae)
Source: PeerJ. 2025 Jan 14;13:e18694. doi: 10.7717/peerj.18694 (PMC11740736; doi:10.7717/peerj.18694)
Supplement: Supplemental Information 9 — Significant results are marked in bold. [file peerj-13-18694-s009.docx]

**S9** Standardized log-odds-ratio (LOR) chi square (*X^2^*) statistics and the associated two-sided-*p*-values to test for “transience” and “trap-dependence” for MARK formulation at both sampling sites and during each sampling period at Malbato. Significant results are marked in bold.

|  |  |  | **Test for Transience** | |  | **Test for Trap-dependence** | |  |
| --- | --- | --- | --- | --- | --- | --- | --- | --- |
| **Sampling site** | **Sampling period** | **Age class** | ***X^2^*** | ***p-value*** | | ***X^2^*** | ***p-value*** | |
| Malbato | All together (2022-2023) | - | 4.172 | **0.003*** | | -4.0935 | **0.004*** | |
|  |  |  |  |  | |  |  | |
| Malbato | April-July 2022 | Adults | 0.757 | 0.449 | | -1.246 | 0.213 | |
|  |  | Subadults | 2.289 | **0.022*** | | -0.051 | 0.959 | |
|  | October-December 2022 | Adults | -0.178 | 0.858 | | -0.406 | 0.684 | |
|  |  | Subadults | 0.444 | 0.656 | | -0.346 | 0.729 | |
|  | April-June 2023 | Adults | 1.716 | 0.086 | | -0.347 | 0.727 | |
|  |  | Subadults | 2.250 | **0.024*** | | 0.198 | 0.842 | |
|  |  |  |  |  | |  |  | |
| San Rafael | All together (2022-2023) | - | 0.889 | 0.373 | | -1.122 | 0.261 | |
