# Supplementary material for: Demography and homing behavior in the poorly-known Philippine flat-headed frog Barbourula busuangensis (Anura: Bombinatoridae)
Source: PeerJ. 2025 Jan 14;13:e18694. doi: 10.7717/peerj.18694 (PMC11740736; doi:10.7717/peerj.18694)
Supplement: Supplemental Information 10 [file peerj-13-18694-s010.docx]

**S10** Output table of the multiple logistic regression model, with age (subadult *vs* adult), distance (10, 30 or 50 m) and direction (upstream or downstream) as predictors and homing success as the dependent variable.

| **Predictor** | **Estimate (B)** | **Standard error** | **Wald** | ***p*** |
| --- | --- | --- | --- | --- |
| Age | 0.849 | 0.514 | 2.732 | 0.098 |
| Distance | -0.530 | 0.273 | 3.782 | 0.052 |
| Direction | 0.042 | 0.469 | 0.008 | 0.928 |

Model statistics: R^2^ = 0.094 (Cox and Snell), 0.126 (Nagelkerke); Model x^2^(1) = 8.071, p < 0.045.
